# Influence of heat-non-burn tobacco aerosol on the microbiome of biofilm from human whole saliva bacteria in vitro

**DOI:** 10.1007/s00784-026-07018-z

**Published:** 2026-07-16

**Authors:** Felix Hellwig, Eva Kohnert, Elmar Hellwig, Fabian Cieplik, Sibylle Bartsch, Jörg Philipp Tchorz, Markus Jörg Altenburger, Ali Al-Ahmad

**Affiliations:** 1 Department of Operative Dentistry and Periodontology, Center for Dental Medicine, Medical Center & Faculty of Medicine, University of Freiburg, Hugstetter Str. 55, 79106 Freiburg, Germany; 2 Institute for Medical Biometry and Statistics, Faculty of Medicine, University of Freiburg, Freiburg im Breisgau, Germany; 3 Division for Endodontics, Center for Operative Dentistry and Periodontology, Department of Dentistry, Faculty of Medicine and Dentistry, Danube Private University, Krems, Austria; 4 Department of Orthodontics, Center for Dental Medicine, Medical Center-University of Freiburg, Faculty of Medicine, Albert-Ludwigs-University of Freiburg, Freiburg im Breisgau, Germany

**Keywords:** Tobacco smoke, Biofilm reactor, IQOS aerosol, Oral biofilm, Oral microbiome

## Abstract

**Objectives:**

The heat-not-burn tobacco product IQOS (I Quit Ordinary Smoking) has recently become widely used. However, the impact of IQOS aerosol on the oral microbiome remains unclear. The present study therefore aimed to investigate the influence of IQOS aerosol on the microbial composition of microcosm biofilms formed from human saliva using a standardized biofilm reactor.

**Material and methods:**

A custom-designed biofilm reactor was constructed to enable the intermittent exposure of biofilms to IQOS aerosol. Microcosm biofilms were formed on bovine enamel samples with a defined surface (19.635 mm^2^) with unstimulated pooled human saliva from three healthy probands being used as inoculum. Biofilm formation took place with continuous nutrient medium supply for 5 days. The biofilm in the test setup was exposed to IQOS aerosol 8 times a day for 5 min each time. A parallel test setup ensured simultaneous biofilm formation without exposure to IQOS aersol and served as a negative control. After 5 days, the microbial composition of the formed biofilms was examined by amplicon sequencing using the V1-V3 region of the 16S rRNA gene. In addition, the biofilm was visualized using scanning electron microscopy.

**Results:**

After one week, the surfaces of the bovine enamel samples on which biofilm formation took place were similarly covered, whether under the influence of IQOS aerosol or in the negative control. The Simpson index showed significant differences (*P* < 0.05), while the Pielou index showed highly significant differences (0.01 < *P* < 0.05), as did the Shannon index (0.001 < *P* < 0.01) and the Richness index (*P* < 0.001). The β-diversity showed different clustering between the treated biofilm and the negative control, corresponding to the significantly different (*p* = 0.001) microbial community caused by the IQOS aerosol. The abundance of the genera *Gemella*, *Haemophilus*, *Neisseria* and *Rothia* was significantly lower in biofilms influenced by IQOS aerosol. Additionally, the abundance of different species was significantly modified by IQOS aerosol.

**Conclusions:**

IQOS aersol may shift oral microbial composition, even though not inhibiting biofilm growth. This highlights the need for further research into the effects on oral microbal ecology of IQOS users, as well as the development of prevention and education measures regarding the potential health risks associated with IQOS.

**Supplementary Information:**

The online version contains supplementary material available at 10.1007/s00784-026-07018-z.

## Introduction

Smoking tobacco is a major health problem and causes the premature death of up to 8 million people worldwide each year [1]. The high mortality rate caused by smoking is due to various diseases such as cancer, respiratory disorders, cardiovascular diseases, chronic obstructive pulmonary disease (COPD), chronic bronchitis, and pulmonary emphysema [2]. In the oral cavity, smoking is a risk factor for oral cancer, gingivitis, periodontitis, halitosis, and tooth discoloration [3, 4]. The smoke from combustible tobacco cigarettes contains a wide variety of harmful substances, including particles with excessive calcium ion concentrations, more than 500 organic compounds, and free radicals, which can cause oxidative stress in human cells [5–7]. In an attempt to minimize the health damage caused by tobacco consumption, a wide variety of non-combustible nicotine products have been developed and brought to market, such as electronic nicotine delivery systems including heated tobacco, e-cigarettes and vaporizers or non-inhaled nicotine products including snus and nicotine pouches [8, 9].

In 2014, Philip Morris International introduced a heat-non-burn tobacco product called IQOS in Japan and Italy to the market. It was approved for the US market by the US Food and Drug Administration (FDA) in 2019 and is now widely used by many tobacco consumers worldwide [2, 10]. The product consists of a holder that heats the tobacco sticks by induction and a pocket that charges the holder. Philip Morris International claimed that their goal was to minimise the emission of harmful substances, thereby reducing the harmful effects of smoking. It was assumed that this goal would be reached by heating the tobacco to a temperature of only 350 °C, as opposed to the temperatures of 640–780 °C reached when consuming classic combustible tobacco cigarettes [11]. However, there are controversial discussions about the toxicity of IQOS aerosol [2]. There is evidence that IQOS aerosol contains toxic substances such as tar, aldehydes (formaldehyde, acetaldehyde) and nitrosamines, which are similar to the ingredients of traditional cigarette smoke, even though their concentrations are lower than in cigarette smoke [12–14].

The oral biofilm consists of more than 700 different types of bacteria that interact with each other in close proximity and, when healthy, exist in a state of homeostasis that benefits both the host and the microorganisms themselves [15, 16]. When this balance is disrupted by certain environmental factors, such as frequent and excessive sugar consumption and the use of disinfectants, dysbiosis of the oral microbiota occurs, leading to oral diseases [17–19]. An important environmental factor that can influence the oral microbiome in the oral cavity is smoking. Tobacco smoke can alter the microbial ecology of the oral cavity through various mechanisms such as antibiotic effects, oxygen deprivation, interaction with the immune system, and changes in the local microenvironment, leading to changes in the colonizability of certain bacteria such as *Streptococcus* spp., *Veillonella* spp., and *Neisseria* spp. [20, 21]. Microbiome studies have shown that the prevalence of various oral disease-associated bacteria such as *Streptococcus mutans*, *Prevotella denticola*, *Treponema* spp., and *Veillonella dispar* increases in cigarette smokers, both in healthy individuals and in patients with mild periodontitis [20]. There is also evidence that aerosols from conventional vapers can affect the composition of the oral microbiota in such a way that the risk of oral diseases such as caries and periodontitis increases [22–25]. However, the effects of IQOS aerosol on the oral microbiome have not yet been studied using a standardized method. The aim of the present study was therefore to investigate the influence of IQOS aerosol on the biofilm microbiome obtained from pooled human saliva bacteria, using a standardised biofilm reactor.

## Material and methods

### Heat-non-burn tobacco product

The IQOS ILUMA device consists of a “pocket” and a “holder.” The pocket serves as a battery for charging the holder. Tobacco sticks are inserted into the holder, which are heated using an induction process (SMARTCORE INDUCTION SYSTEM™) and produce nicotine-containing aerosol. The tobacco sticks consist of a seal, a heating element, tobacco, a hollow channel, and a cooling segment. These parts contain the materials paper, cellulose acetate, triacetin metal (stainless steel), and deep adhesive. A tobacco stick can be consumed for up to 6 min or switches itself off after 12/14 puffs. When the tobacco stick is inserted, electricity flows, creating a magnetic field that heats the metal inside the tobacco stick to approximately 350 degrees. This produces nicotine-containing aerosol, which is inhaled. The nicotine content is the same in all tobacco sticks and is 0.5 mg. A variety of organic compounds are present in the aerosol, including polycyclic aromatic hydrocarbons, glycerin and propylene glycol.

### Biofilm reactor and experimental design

For the experiment, bovine enamel samples (BES) with a thickness of 1 mm and a diameter of 5 mm and thus an enamel surface area of 19.635 mm^2^ were used, which were obtained from incisors of freshly slaughtered BSE (bovine spongiform encephalopathy)-free cattle. The preparation of bovine enamel samples has been described in detail previously [26, 27]. In brief, the BES were prepared using a trephine bur (custom fabrication; Komet Dental, Gebr. Brasseler GmbH & Co.KG, Lemgo, Germany). Bovine teeth were obtained from the local slaughter house. Use of the tissues was approved by the local veterinary authority (City of Freiburg im Breisgau; Office for Public Order – Veterinary Department, AZ: 32.602.01, June 19th, 2024). The surface of the BES was sanded down to a grit size of 4000 using waterproof silicon carbide paper (Struers GmbH, Ballerup, Denmark) on a wet disc grinder (Knuth-Rotor 3, Struers GmbH, Ballerup, Denmark). The BES were then disinfected in a NaOCl solution (3%) in an ultrasonic bath, followed by a second disinfection in ethanol (70% v/v). The BES were then treated twice in double-distilled water for 5 min each time in an ultrasonic bath to remove the remaining NaOCl and ethanol.

**Fig. 1:**
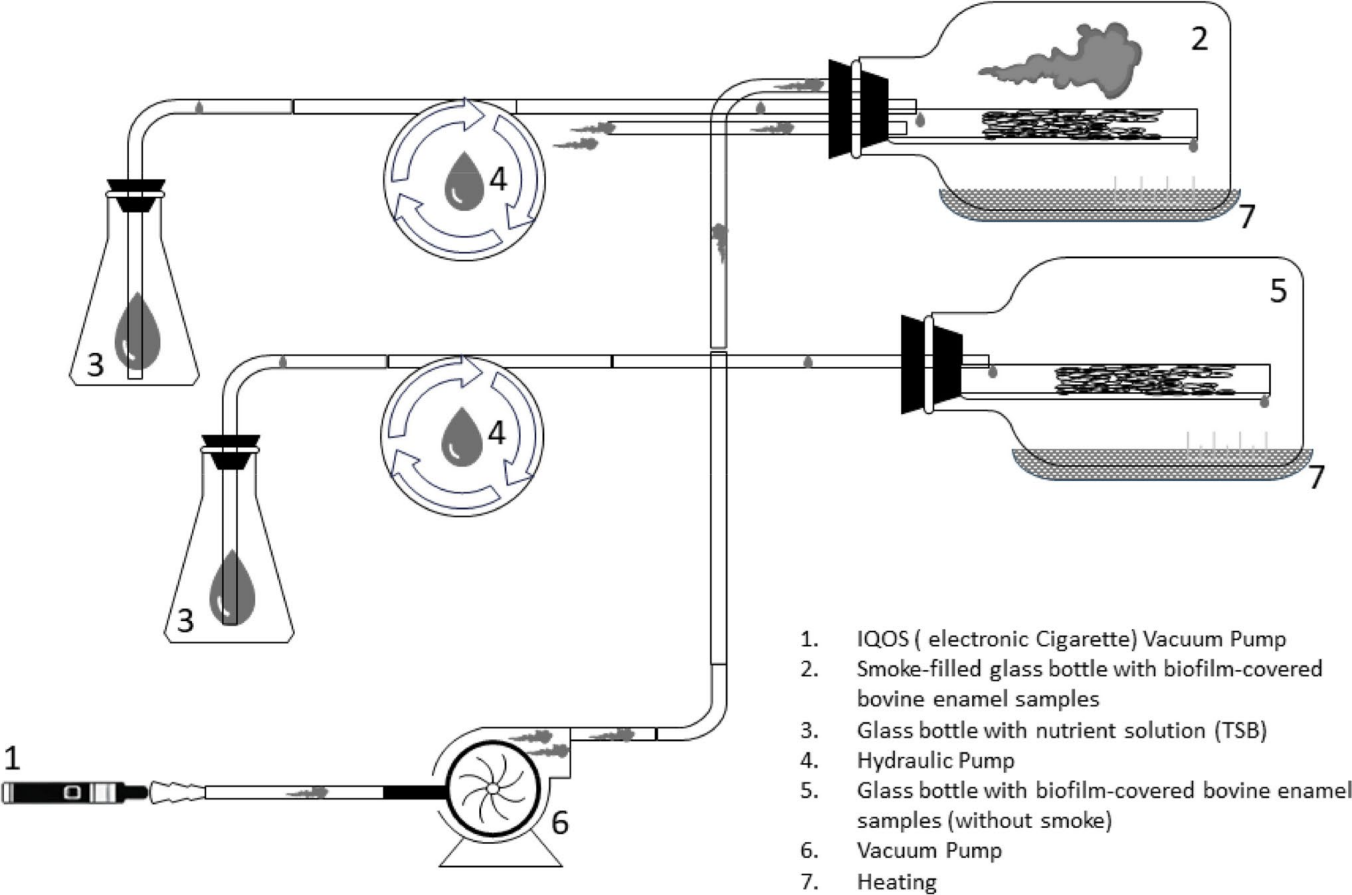
Schematic representation of the experimental setup and biofilm reactor used. The experimental setup enables standardized investigation of the effect of IQOS aerosol on biofilm formation in vitro

A custom-designed biofilm reactor was constructed to enable the intermittent exposure of cultivated biofilms to IQOS aerosol from the beginning of the experiment. Specimen holders were fabricated using a filament-based 3D printer and polylactic acid (PLA) (both: Ultimaker Zaltbommel, The Netherlands) (see Fig. 1). In addition to the specimen holder, the screw cap was equipped with an inlet for culture medium, allowing the medium to drip onto the specimen holder while preventing back-contamination. The culture medium then flowed from the specimen holder into a bottle positioned nearly horizontally. To simulate the act of smoking, a pump—calibrated to mimic the inhalation force of a human lung—was connected to the outlet of the bottle, thereby generating negative pressure. The intake was linked to the heat of the IQOS device. Unstimulated human pooled saliva from three adults was collected in a sterile falcon tube and used as inoculum. The Ethics Committee of the Albert-Ludwigs-University Freiburg approved the use of unstimulated human saliva (No. 23–1537-S1-AV). 12 BES were then incubated with the unstimulated pooled human saliva to allow the initial microbial adhesion, then placed in special glass containers (4,7) and secured to the lid. This was conducted in quadruplicate, with 48 BES used to cultivate the biofilm across four lanes for each experiment. The bottle was kept at a temperature of 37 °C using a water bath. The BES covered by initially adhered oral bacteria and fixed in the biofilm container of the biofilm reactor were supplied with nutrient solution (5, TSB) via syringe attachments and peristaltic pump for 5 days to cultivate biofilm simulating the formation of mature oral biofilm. The nutrient solution consists of tryptones, soy peptone, sodium chloride, glucose, and dipotassium phosphate. The nutrient solution was continuously applied to the enamel samples by a peristaltic pump (6) (flow rate protocol: 1 min 1 ml/min; 5 min no flow). In one biofilm container, a vacuum pump (3), which was intended to simulate lung breathing, was used to draw on the externally attached IQOS (1) and periodically pass aerosol for five minutes over the enamel samples. This process took place eight times a day each one hour over a period of one week, from Monday to Friday. No aerosol was passed over the BES in the second biofilm container which served as negative control. The BES covered with the cultured biofilm were then removed from the biofilm containers and prepared for further analysis. Three BES from each lane, containing 12 BES from the negative control and test biofilms, were used to visualise the cultured biofilms using a scanning electron microscope. Microbiome analysis of the residual nine biofilm samples from each lane and biofilm container was performed to evaluate the effects of IQOS aerosol on biofilm formation. The experiment was conducted twice, resulting in eight sequenced biofilm samples from the test container exposed to IQOS and eight from the negative control.

### Analysis of the formed biofilm by scanning electron microscopy (SEM)

Three biofilm samples (3 BES) from the biofilm container exposed to IQOS aerosol and from the negative control, each, were stored in 8% formaldehyde for at least two days at 4 °C. The fixed biofilm samples were then dehydrated in an ascending series of ethanol (50,70,80,90%) and twice in 100% ethanol, followed by drying in a critical point dryer (CPD 030, BAL-TEC AG, Pfäffikon, Switzerland). The BES were then sputter-coated with gold using a fine coater (JFC-1200 fine coater, Jeol GmbH, Freising, Germany). SEM Images of different magnifications were prepared at 10 kV from all BSE (JSM-IT100 In TouchScope, Version 1.090, Jeol GmbH, Freising, Germany).

### DNA isolation and amplicon sequencing

The DNeasy Blood and Tissue Kit (Qiagen, Hilden, Germany) was used to extract the DNA as described before [17]. As positive control an own mock community was used as described by Anderson et al. [28], with the following microbial species: *Actinomyces odontolyticus* (DSM 19120), *Neisseria flavescens* (DSM 17633), *Fusobacterium nucleatum* (ATCC 25586), *Streptococcus mutans* (DSM 6178), *Streptococcus sanguinis* (DSM 20068), *Streptococcus mitis* (ATCC11843), *Porphyromonas gingivalis* (W381), *Veillonella parvula* (DSM 2008), *Parvimonas micra* (ATCC 33270), and *Tannerella forsythia* (ATCC 43037). DNA amplicon sequencing was performed using the V1-V3 region of the 16S rRNA gene sequence (Eurofins Genomics, Konstanz, Germany). The sequencing data generated in this study are available in the European Nucleotide Archive (ENA) under project accession number PRJEB111005.

### Bioinformatics

Paired-end 16S rRNA gene sequencing data were processed using the QIIME2 framework. Raw sequence data, provided in FASTQ format, were first imported into QIIME2 as a.qza artifact using the qiime tools import function. Sequence quality was assessed with the qiime demux summarize function. The DADA2 algorithm (qiime dada2 denoise-paired) was employed for quality filtering, chimera removal, and paired-end sequence denoising, with forward reads truncated at 291 bp and reverse reads truncated at 268 bp due to a rapid drop off of the quality score. A feature table was generated and summary statistics were computed using the qiime feature-table summarize and qiime feature-table tabulate-seqs commands.

For phylogenetic analysis, sequences were aligned using MAFFT, and a phylogenetic tree was constructed with FastTree via the qiime phylogeny align-to-tree-mafft-fasttree pipeline. Taxonomic classification was performed using the SILVA 138 reference database. A custom classifier was trained using the qiime feature-classifier fit-classifier-naive-bayes function on the SILVA 138 99% OTU reference sequences, with amplicons extracted based on the forward (AGRRTTYGATYMTGGCTCAG) and reverse (TBACCGCGGCTGCTGGCAC) primers. Classification of sequences was then carried out with the trained classifier using the qiime feature-classifier classify-sklearn function.

### Downstream analysis and statistical analysis

All downstream analyses were conducted in R-Studio (R version 4.3.3). Data was imported into a phyloseq object from the phyloseq R-package. Results are presented at the species level. Additionally, a genus and phylum level data set was created by agglomerating the data at genus and phylum level. For all statistical tests a significance level of 5% was applied.

To characterize bacterial communities within a sample α-diversity was analyzed. The Shannon-Weiner diversity index, InvSimpson, Richness and Pielou’s evenness index were calculated as a measure for α-diversity using the vegan package. Results are presented as boxplots using the ggplot2 package. To test for differences in alpha diversity between conditions a linear mixed effects model was employed, taking the paired nature of the data into account and adjusting for batch effects of different experiments.

Microbial similarity between samples was investigated using β-diversity. The phyloseq package was used to calculate Bray–Curtis distances for dissimilarity measurement and to plot the results using non-metric multidimensional scaling. A pairwise PERMANOVA with 999 permutations based on the adonis function from the vegan package was applied to test for differences in β-diversity between timepoints.

Within the MaAsLin2 framework, a mixed-effects model was employed to account for the paired nature of the data. This model was used to identify species and genera that were differentially abundant between conditions, adjusting for batch effects of different experiments. A 10% prevalence threshold was applied, and the data were LOG-transformed. To control for multiple comparisons, the Benjamini–Hochberg correction was applied. For all taxa that showed significant changes, boxplots were generated using the ggplot2 package for visualization.

## Results

In the present study the effects of aerosol produced by a heat-non-burn tobacco device (IQOS) on biofilm formation by total human salivary bacteria was studied using a standardized biofilm reactor. After the initial microbial adhesion, the biofilm formation was exposed to IQOS aerosol for specific time intervals over 8 h per day for a total duration of one week. Simultaneous biofilm formation without exposure to IQOS aerosol served as negative control. The diversities and (dis-)similarities of the microbiome was analyzed using DNA amplicon sequencing of the 16S rRNA gene. Additionally, the formed biofilm was studied by scanning electron microscope (SEM).

### Scanning electron microscopic analysis

**Fig. 2:**
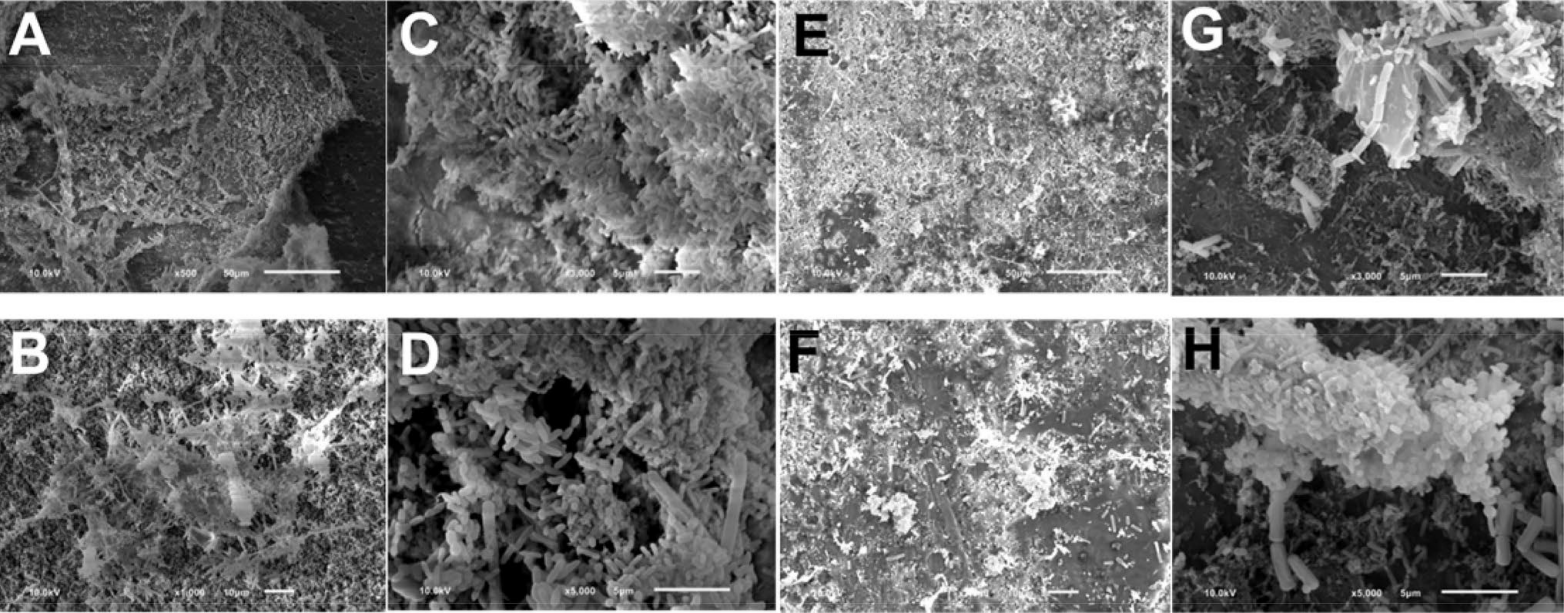
Representative scanning electron microscopy (SEM) images of biofilms formed in the presence of IQOS aerosol (**A**-**D**) and negative control biofilms (**E**–**H**)

Representative SEM images of the biofilm formed under the influence of IQOS aerosol (Fig. 2A-D) and the biofilm from the negative control group (Fig. 2E-H) are shown. Different magnifications were selected to show the morphology of the biofilm formed. Overall, the SEM images showed that IQOS aerosol did not prevent biofilm formation. The surfaces of the bovine enamel samples on which biofilm formation took place were similarly covered after one week, both under the influence of IQOS aerosol and in the negative control group. The SEM images did not reveal any differences in the coverage rate of the biofilm. Furthermore, all biofilms exhibited different morphologies. The cocci-shaped structure of the biofilm bacteria was striking, but rod-shaped bacteria were also visible in all biofilms.

### Microbial diversity

**Fig. 3:**
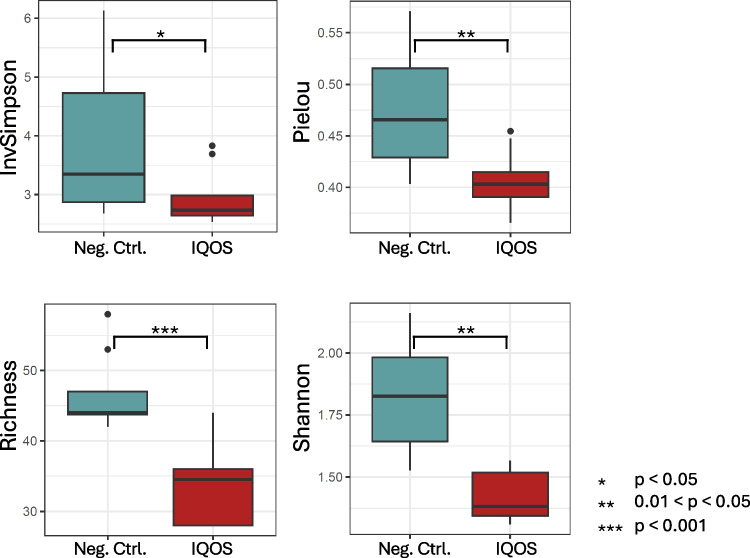
Boxplots of the α-diversity describing the species in the biofilm affected by IQOS aerosol and in the negative control (Neg. Ctrl.) as calculated by four different indices, the InvSimpson index, the Pielou index, the Shannon index, and the Richness index. The boxplots show the minimum, 25th percentile, median, 75th percentile, and maximum of the data from bottom to top

The α-diversity describes the species richness and evenness in the biofilm affected by IQOS aerosol and in the negative control and was calculated using four different indices (Fig. 3). The microbiome of the biofilm formed under the influence of IQOS showed significantly lower α-diversity than the negative control, in which the biofilm was formed without exposure to IQOS aerosol. The InvSimpson index showed significant differences (*P* < 0.05), while the Pielou index (0.01 < *P* < 0.05), Shannon index (0.001 < *P* < 0.01), and Richness index (*P* < 0.001) showed highly significant differences (Fig. 3). These results show that IQOS decreased the richness as well as the evenness of the formed biofilm.

**Fig. 4:**
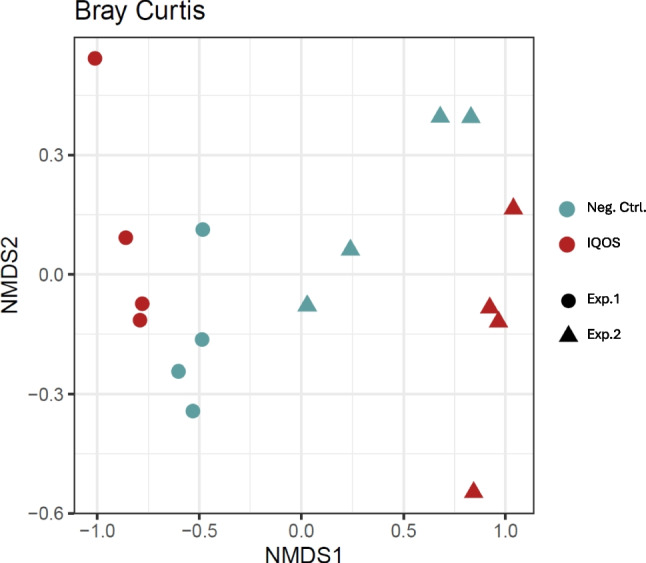
β-diversity depicted by non-metric multidimensional scaling (NMDS) plots based on Bray–Curtis index. A significantly different clustering of the biofilm affected by IQOS aerosol is shown compared to the negative control (Neg. Ctrl.)

The differences and similarities of the microbiota between the biofilm affected by IQOS aerosol and the negative control was characterized by calculating the β-diversity based on the Bray–Curtis distance (Fig. 4). The non-metric multidimensional scaling (NMDS) plots are depicted in Fig. 4 to identify clustering patterns of the biofilm samples revealing dissimilarities in the treated biofilm and the negative control. The β-diversity showed different clustering between the treated biofilm and the negative control which correspond the significantly different (*p* = 0.001) microbial community caused by IQOS aerosol (Fig. 4).

### Microbial community composition

**Fig. 5:**
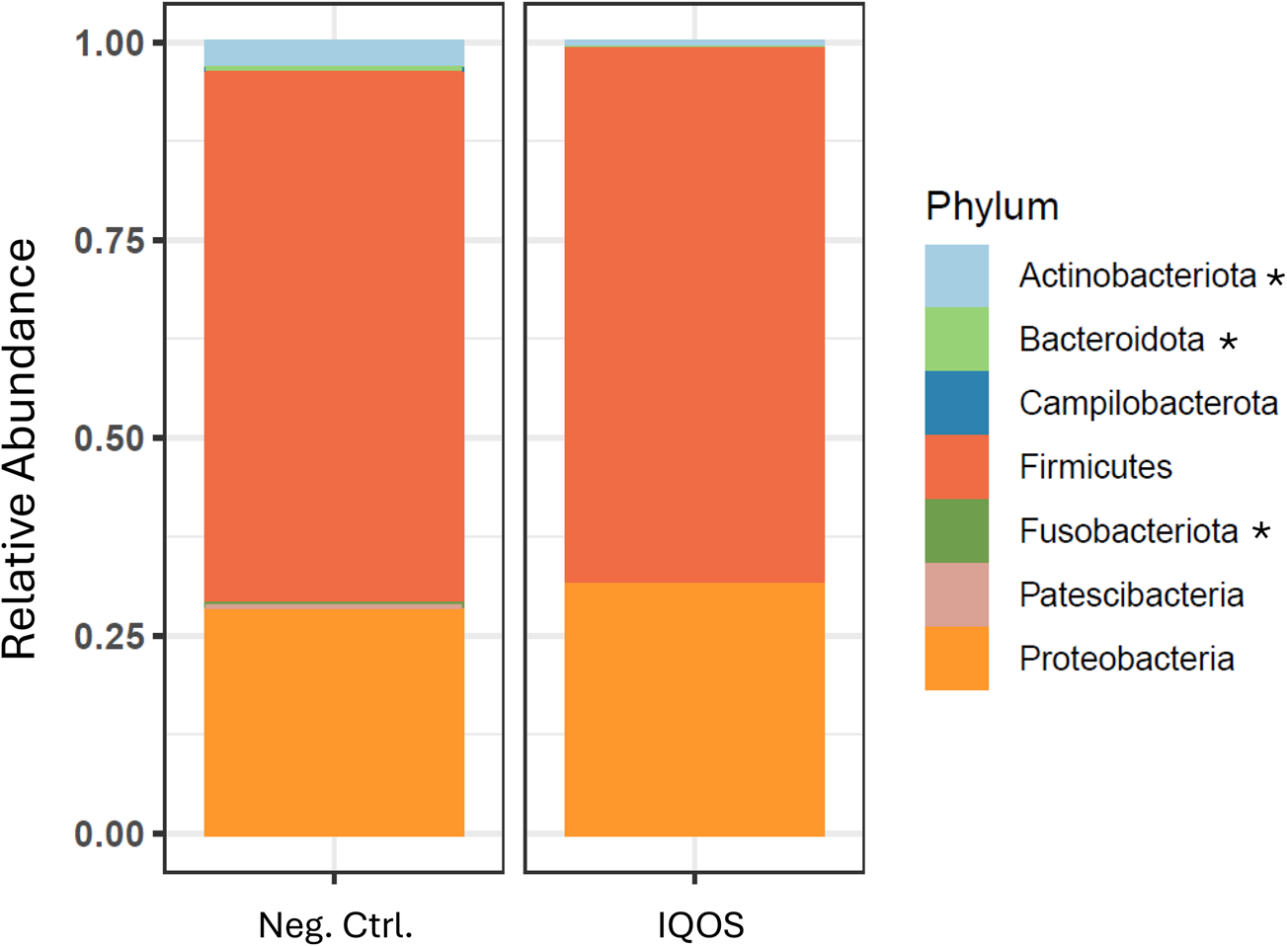
Phyla found in the biofilm affected by IQOS aerosol as well as in the negative control (Neg. Ctrl.)

**Fig. 6:**
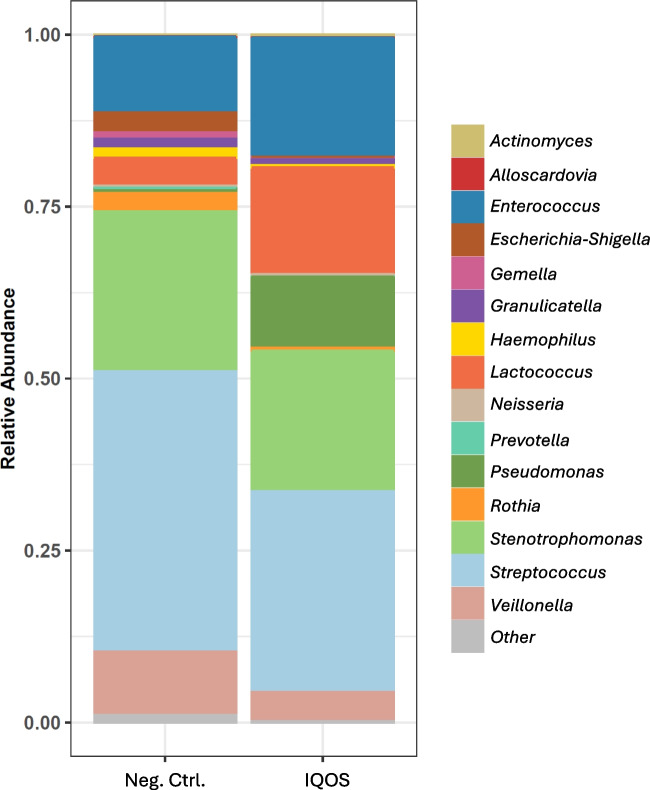
Genera found in the biofilm affected by IQOS aerosol as well as in the negative control (Neg. Ctrl.)

**Fig. 7:**
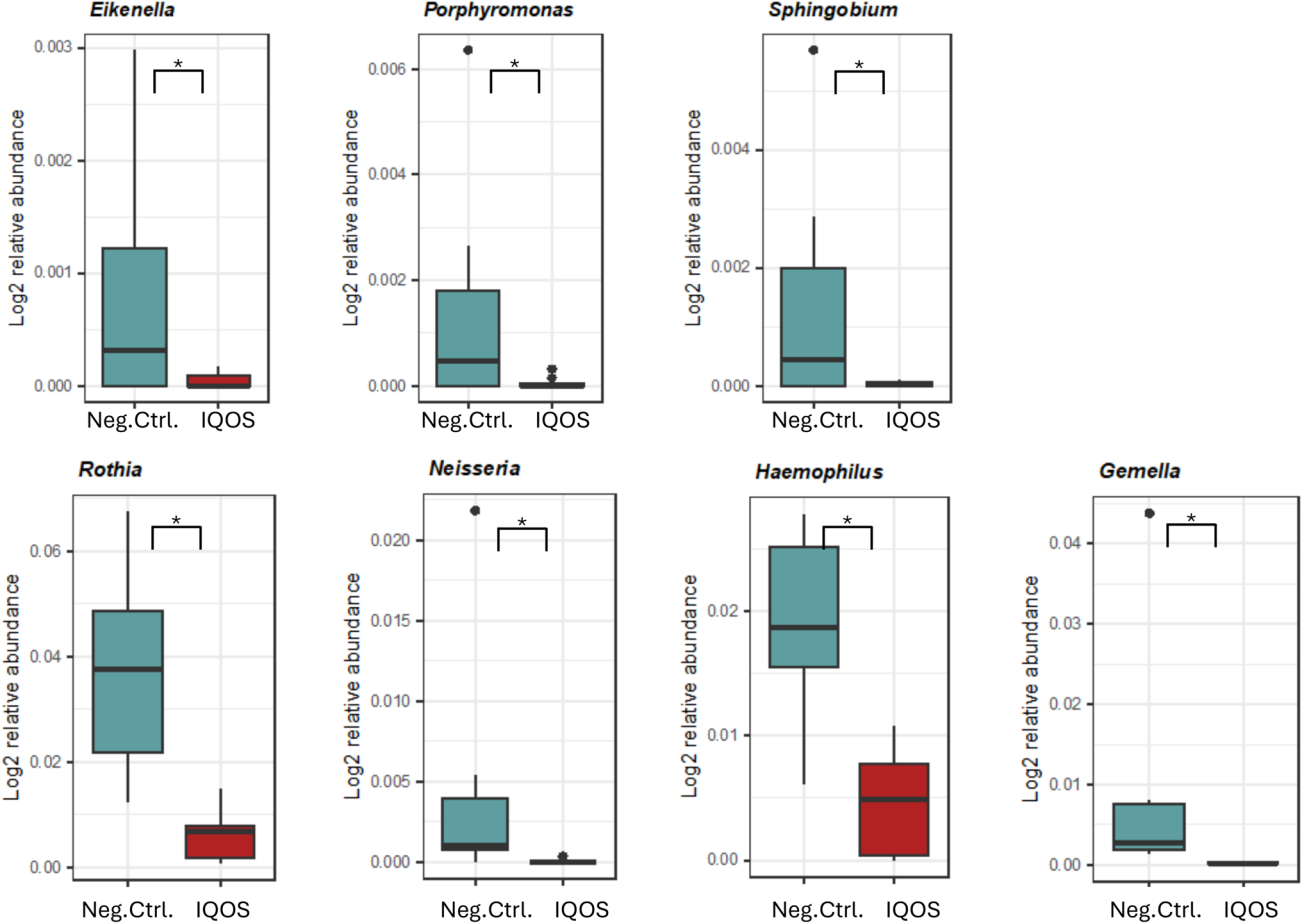
Genera with significant differences between them in the biofilm affected by IQOS aerosol, as well as in the negative control (Neg. Ctrl.)

**Fig. 8:**
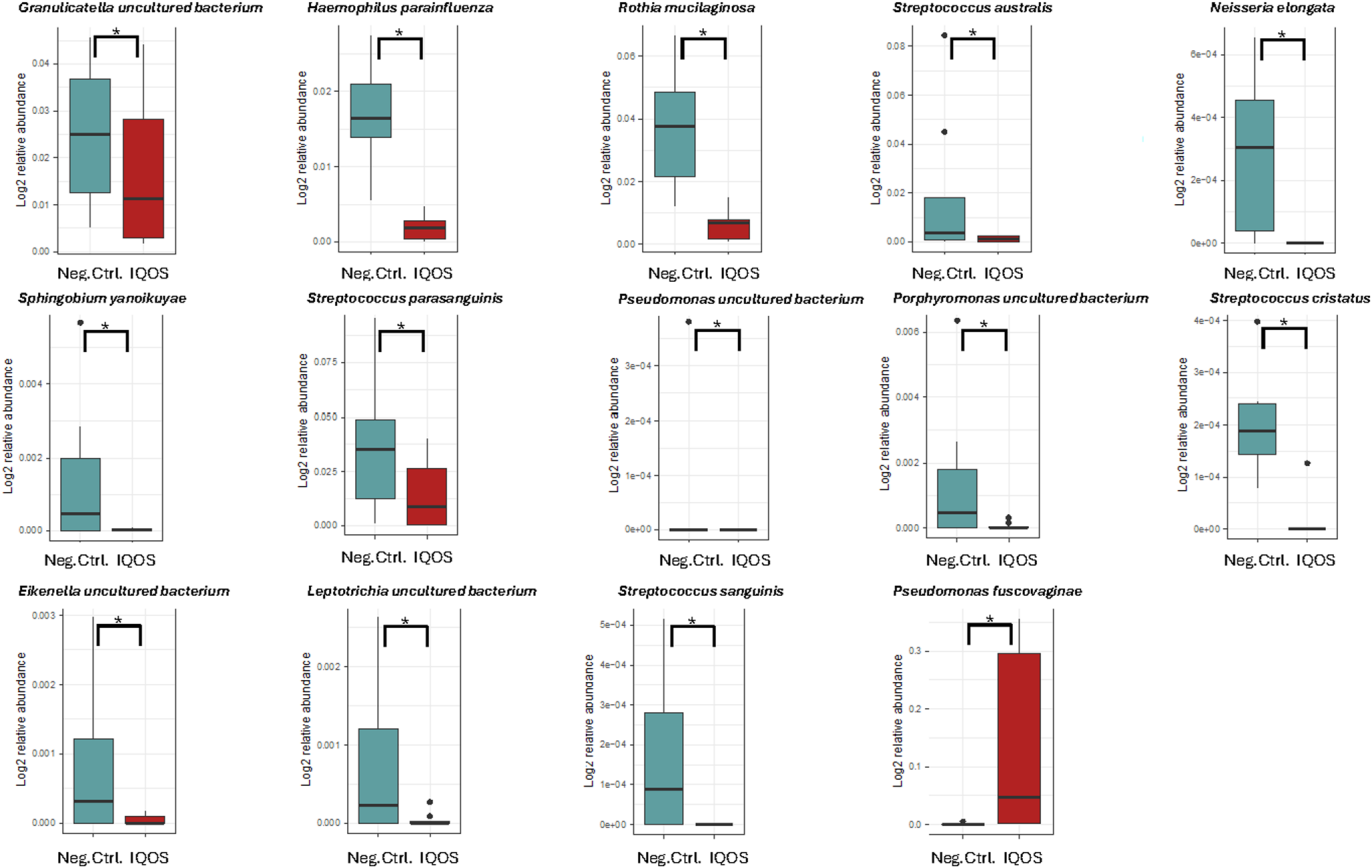
Species with significant differences between them in the biofilm affected by IQOS aerosol, as well as in the negative control (Neg.Ctrl.)

Overall, 53 different genera and 108 species were revealed. All taxa of the mock community, which was used as positive control for DNA extraction, were detected in both the treated and untreated biofilm (Supplementary Figure S1). All detected taxa belonged to the following seven phyla: Actinobacteriota, Bacteroidota, Campylobacterota, Firmicutes, Fusobacteria and Proteobacteria. As shown in Fig. 5 the phyla Actinobacteriota, Bacteroidota, and Fusobacteria were significantly decreased (*P* < 0.05) in the biofilm affected by IQOS aerosol as compared to the negative control. The most frequently detected genera in both biofilms were *Actinomyces*, *Alloscardovia*, *Enterococcus*, *Escherichia-Shigella*, *Gemella*, *Granulicatella*, *Haemophilus*, *Lactococcus*, *Neisseria*, *Prevotella*, *Pseudomonas*, *Rothia*, *Stenotrophomonas*, *Streptococcus*, and *Veillonella*. Of these genera, *Gemella*, *Haemophilus*, *Neisseria*, and *Rothia* were significantly lower in the biofilm influenced by IQOS aerosol (Figs. 6 and 7). Other genera that were not among the most abundant but showed significant differences between the treated and untreated biofilm were *Eikenella*, *Porphyromonas*, and *Sphingobium* (Fig. 7). Of all the species detected, the prevalence of the following species was significantly lower (*P* < 0.05) in the biofilm affected by IQOS (Fig. 8): *Haemophilus parainflunzae*, *Rothia mucilaginosa*, *Streptococcus australicus*, *Neisseria elongata*, *Sphingobium yanoikuyae*, *Streptococcus parasanguinis*, *Streptococcus cristatus*, *Streptococcus sanguinis*, *Granulicatella* sp. (uncultured), *Porphyromonas* sp. (uncultured), *Eikenella* sp. (uncultured), *Pseudomonas* sp. (uncultured). In contrary, the prevalence of *Pseudomonas fuscovagina* was significanty increased in the biofilm formed during exposure to IQOS aerosol (Fig. 8).

## Discussion

While the dysbiotic effect of smoke from traditional cigarettes and aerosols from conventional vapers on the oral microbiota has been the focus of current research [19–25], the possible effects of aerosols from heat-not-burn tobacco products on the oral microbiome have not been investigated so far. In this study, the effects of IQOS, a heat-not-burn tobacco product widely used among consumers, on the microbial composition of a microcosm biofilm cultivated in a standardized biofilm reactor using an inoculum of human pooled saliva were investigated. While the formation of biofilms was unaffected by the IQOS aerosol, it was demonstrated that this aerosol significantly altered the overall diversity of the microbiome. The prevalence of certain phyla, genera, and species of the biofilm formed was either significantly increased or decreased by the IQOS aerosol.

The biofilm reactor used was standardized so that only the additional IQOS aerosol was considered as an effect factor, while all other conditions were kept constant. Similar biofilm reactor designs have been successfully used by our group to test the effects of preservatives on the biofilm of certain oral streptococci or to examine the ability of foreign germs to integrate into a biofilm of total saliva bacteria [29, 30]. Such biofilm reactors do not reflect the complex conditions of biofilm formation in the human oral cavity, where saliva with its various components serves as a nutrient medium. Nevertheless, the biofilm reactor used represented a continuous culture with a permanent continuous nutrient supply rate. Such biofilm reactors are often described in literature and are considered an effective model for in situ biofilm formation [31, 32]. Another advantage of the experimental design used is that biofilm formation occurred during exposure to a certain amount of IQOS aerosol. If the effects of IQOS aerosol on oral biofilm formation were to be considered in vivo, it would be necessary to specify approximate ranges for exposure, as consumption behavior varies from person to person [33], which would mean that a large number of IQOS consumers would be needed to reflect the real situation. Using a defined volume of aerosol and measuring the chemical composition of the used aerosol would have added more impact to the standardisation of the experimental setup used. Nevertheless, the IQOS product has been comprehensively described regarding the chemical composition of its aerosol and the temperature reached when heating the tobacco. In addition, biofilm formation in situ could vary depending on the dietary habits of the test subjects. Such marginal effects could, of course, be minimized by including a very large cohort. Since unstimulated human saliva is a continuous source of nutrients for biofilm formation in the oral cavity, the question arises as to the influence of the complex culture medium used in this study on the composition of the cultured biofilm. Nevertheless, the results of the present study showed a high diversity in the cultured biofilm with phyla, genera, and species typical of oral biofilm. Scanning electron microscopy was used to visualize the biofilm. This method has been used in many studies to investigate biofilm formation and is considered sufficient to show the extent of biofilm formation on surfaces while maintaining the actual coverage rate [34], even though only the morphology of the top layer of the three-dimensional biofilm is visible [35]. SEM was complemented by analysis of the microbiome using NGS via 16S rRNA amplicon sequencing to investigate the influence of IQOS aerosol on the composition of the biofilm formed. This NGS analysis of the microbiome is culture-independent and has the advantage of identifying uncultivable bacteria, as only some of the bacteria actually present in a microbiological niche can be cultivated on available agar plates using culture techniques [36, 37]. Although this method has been described in a plethora of studies on oral biofilms, only very few studies have used an internal standard from a so-called mock community of representative oral bacteria. In the present study, a mock community of 10 different Gram-negative and Gram-positive bacteria compiled by our group and described in earlier studies was used [18, 28]. Since all bacterial species in this mock community could be detected by NGS analysis, the efficiency of the DNA extraction, universal 16S rRNA PCR, and sequencing methods used in the present study were demonstrated.

To date, the influence of IQOS aerosol on the biofilm formation of oral bacteria has not been investigated either in vitro or in situ. Only the effects of leachates from butts, which are produced after the use of IQOS and consist of the used stick and tobacco, on primarily non-pathogenic microorganisms have been investigated in order to evaluate the ecotoxicity of IQOS waste products [38]. The authors found strong inhibitory effects on the growth of the environmental Gram-negative *Delftia acidovorans* and Gram-positive *Staphylococcus warnerii*. However, the results of the aforementioned study are not comparable with the results obtained in the present study, which do not indicate any growth-inhibiting effect of IQOS aerosol on the biofilm formation of total salivary bacteria in vitro. Baran et al. tested leachates from concentrated ingredients of butts resulted from IQOS consumption for their inhibition of planktonic bacteria in liquid culture [38], while the present study evaluated the effects of IQOS aerosol on biofilm formation. Bacteria in biofilms are well-known to be many times more tolerant to environmental stress and antimicrobials than their planktonic counterparts [39].

The SEM images suggest that the IQOS aerosol has no growth-inhibiting effect during biofilm formation in the biofilm reactor. However, additional quantitative methods, such as crystal violet staining, dry CFU (colony-forming units) counting or volumetric analysis by confocal microscopy, are required to confirm these results. Additionally, it should be noted that the duration of the experiment (5 days) is rather too short to compare this with the results for smokers in the literature. This is because studies of oral biofilm in smokers provide results after several years of smoking. This is, of course, a limitation of the present study. The literature reports that traditional cigarette smokers and e-cigarette smokers have a higher biofilm accumulation rate than non-smokers [40–43]. However, the oral hygiene habits of smokers and non-smokers must not be ignored, as the behavior of the subjects with regard to oral hygiene can distort the results of the plaque index. One limitation of this study is that it is unable to draw comparative conclusions regarding the growth-promoting effects of traditional and e-cigarette consumption that have already been reported. To investigate this comparatively, long-term IQOS consumers must be examined for oral health parameters. An additional limitation of the present study is that the biofilm model cannot simulate the real complex situation within the oral cavity, including the different nutrients derived from the complex and diverse composition of human saliva, compared to the culture medium used, which definitely modifies the growth of the biofilm in the reactor. Nevertheless, the present study achieved standardisation of the approach used, and the high diversity of the cultivated biofilm in the negative control showed that many important members of the oral microbiota could grow in the biofilm reactor used. Nevertheless, the SEM images do not indicate any growth-inhibiting effect of the IQOS aerosol during biofilm formation in the biofilm reactor.

In the present study, the diversity and richness of the biofilm formed during exposure to IQOS aerosol after 5 days were reduced compared to the negative control. These results are consistent with those of Yu et al. [44], who analyzed the microbiome of buccal swab samples from 20 smokers of conventional cigarettes and compared it with the microbiome of samples from non-smokers. However, the authors did not find any clear change in diversity for samples from other sites in the oral cavity. The authors concluded that smoking affects different niches in the oral cavity differently. A study by Lin et al. [45] also showed a significant reduction in the diversity of the salivary microbiome of 30 smokers of conventional cigarettes compared to non-smokers. The results of our study are also consistent with the results of an earlier study that compared the microbiome of the tongue coating of 144 conventional cigarette smokers with that of non-smokers [46]. The authors found a significant reduction in the diversity and richness of the tongue coating microbiome in smokers. A significant reduction in richness was also reported for mixed mucosal samples from the oral cavity in cigarette smokers as well as in smokers and regular alcohol drinkers [47]. However, there are studies that found no change in the diversity or richness of the oral microbiome in saliva samples [48] or supragingival samples [49] from cigarette smokers compared to non-smokers. There are even studies that have shown an increase in the diversity or richness of the oral microbiome in saliva samples or mouthwash samples from cigarette smokers compared to non-smokers [50–52]. The discrepant results in the literature could be due to the heterogeneity of the respective study cohorts and the difficulties in standardizing sample collection. A study of the influence of traditional cigarette smoke and IQOS aerosol on the microbiome of whole human saliva bacteria under standardized biofilm reactor conditions, as reported in the present study, has not yet been conducted.

All phyla detected in the present study except Campylobacterota as well as most of the detected genera have been frequently detected in the oral cavity [17, 18, 28]. Representatives of Campylobacterota have also been reported in the saliva microbiome in a few studies [53, 54]. This shows that the biofilm formed in the biofilm reactor contains a cross-section of oral bacteria. Compared to the oral cavity, a selection is to be expected. Nevertheless, the results show that the model provided proof of concept for the influence of IQOS aerosol on saliva bacterial biofilm. In the present study the phyla Actinobacteriota, Bacteroidota, and Fusobacteria as well as the genera *Gemella*, *Haemophilus*, *Neisseria*, and *Rothia* were significantly decreased. This suggests that IQOS aerosol may have a modifying effect on the oral microbiome. The literature reports inconsistent results regarding the influence of traditional cigarette smoke and e-cigarettes [40, 50]. Mason et al. reported a lower prevalence of *H. parainfluenzae* and *Streptococcus sanguinis* in the subgingival microbiome of smokers, which is consistent with the results of the present study [55]. As shown in the present study, other studies reported lower levels of Proteobacteria phylum members [56, 57]. Lower prevalences of the genera *Neisseria* and *Haemophilus* were also reported in the oral microbiome of smokers [46, 52, 58]. The reduction in the prevalence of important commensal genera such as *Gemella*, *Haemophilus*, *Neisseria*, and *Rothia* suggests a negative effect of IQOS aerosol on the oral microbiome, as has been reported for traditional cigarette smoke and e-cigarette aerosol [20–25]. In particular, the reduction in the prevalence of nitrate-reducing genera *Neisseria* and *Haemophilus* should be further investigated in additional studies using in situ biofilm samples from the oral cavity of IQOS users, as nitrat reduction has a particularly beneficial effect on health [59]. Nevertheless, the results of the microbiome analysis should be considered with caution in terms of their clinical significance. Additional computational metabolic analysis of the detected taxa is required to evaluate the impact of the taxonomic changes revealed by amplification sequencing of the 16S rRNA genes in the present study. Furthermore, future studies should incorporate metagenomic sequencing methods, such as shotgun analysis, to draw functional conclusions alongside the taxonomic analysis.

In conclusion, despite the limitations of this in vitro study, it can be stated that IQOS aersol may have a potentially modifying effect on the microbial composition of microcosm biofilms formed from pooled human saliva, even though biofilm growth was not inhibited. However, future studies should evaluate the impact of changes in the microbiome exposed to IQOS on the mutualistic interactions of bacteria within biofilms, and consequently on bacterial viability, metabolic activity, acid production and total biomass, in order to comprehensively evaluate the effect of IQOS aerosol on the total ecology of the oral microbiota. This underscores the need for further studies on the oral microbiome of IQOS users, as well as on prevention and education measures regarding the possible negative health aspects of IQOS.

## Supplementary Information

Below is the link to the electronic supplementary material.

Supplementary file1 (PDF 501 KB)

## Data Availability

The sequencing data generated in this study are available in the European Nucleotide Archive (ENA) under project accession number PRJEB111005.
