# Supplementary figures and images for: Influence of heat-non-burn tobacco aerosol on the microbiome of biofilm from human whole saliva bacteria in vitro
Source: Clin Oral Investig. 2026 Jul 16;30(8):338. doi: 10.1007/s00784-026-07018-z (PMC13375841; doi:10.1007/s00784-026-07018-z)

Figure S1: Composition of the used mock community.

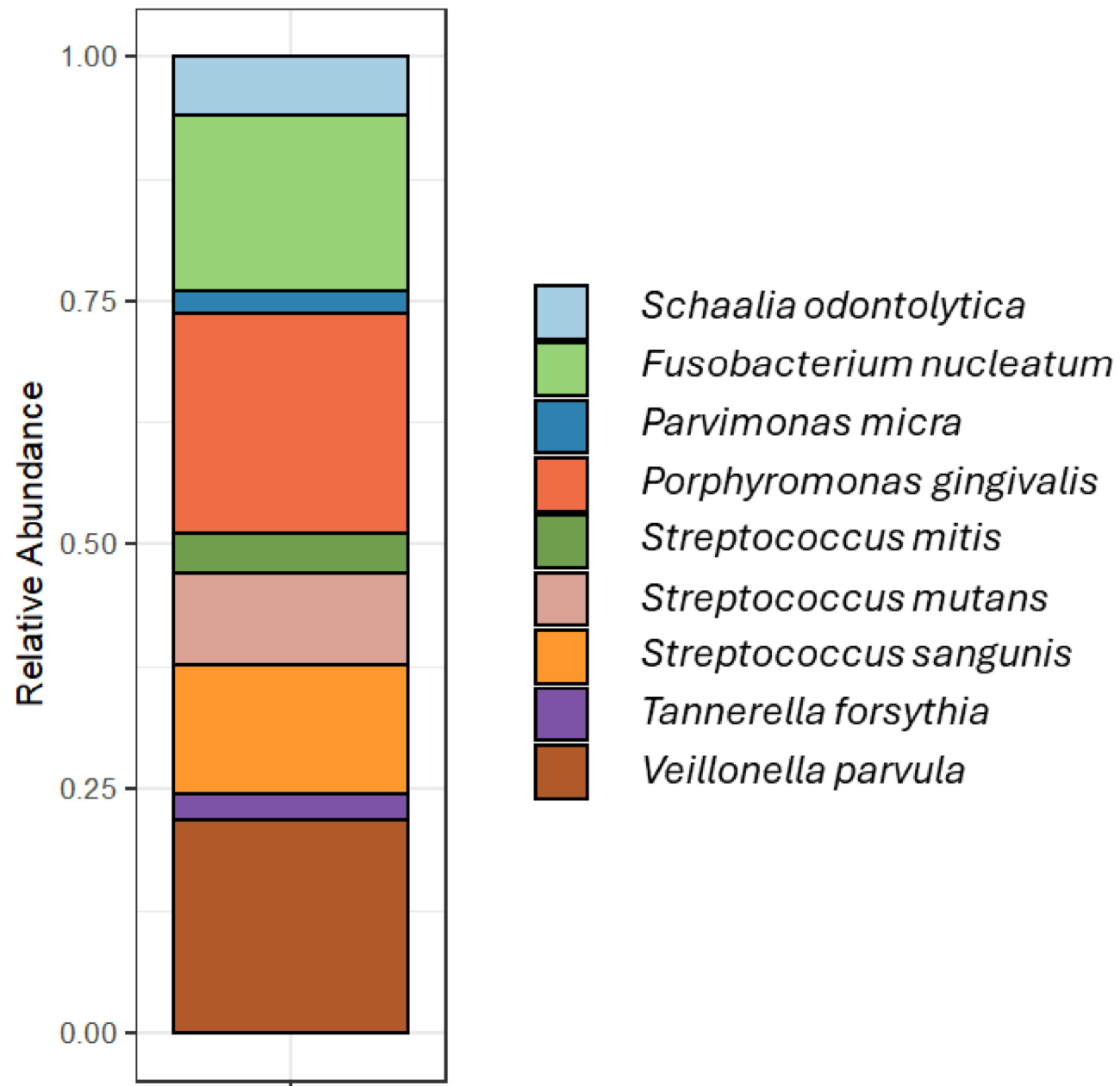

Supplement: Supplementary file 1 — Supplementary file1 (PDF 501 KB) [file 784_2026_7018_MOESM1_ESM.pdf]
